# Chemogenetic Activation of CX3CR1-Expressing Spinal Microglia Using Gq-DREADD Elicits Mechanical Allodynia in Male Mice

**DOI:** 10.3390/cells10040874

**Published:** 2021-04-12

**Authors:** Fumihiro Saika, Shinsuke Matsuzaki, Shiroh Kishioka, Norikazu Kiguchi

**Affiliations:** 1 Department of Pharmacology, Wakayama Medical University, 811-1 Kimiidera, Wakayama City, Wakayama 641-0012, Japan; fumifumi@wakayama-med.ac.jp (F.S.); shinsukemtszk@gmail.com (S.M.); 2 Faculty of Wakayama Health Care Sciences, Takarazuka University of Medical and Health Care, Wakayama City, 2252 Nakanoshima, Wakayama 640-8392, Japan; kishioka@wakayama-med.ac.jp; 3 School of Pharmaceutical Sciences, Wakayama Medical University, 25-1 Shichibancho, Wakayama City, Wakayama 640-8156, Japan

**Keywords:** dorsal horn, cytokine, chemokine, inflammation, neuropathic pain

## Abstract

It is important to investigate the sex-dependent roles of microglia in pain hypersensitivity as reactive microglia within the spinal dorsal horn (DH) have been reported to be pivotal in neuropathic pain induction in male rodents upon nerve injury. Here, we aimed at determining the role of sex differences in the behavioral and functional outcomes of the chemogenetic activation of spinal microglia using Gq-designer receptors exclusively activated by designer drugs (Gq-DREADD) driven by the microglia-specific Cx3cr1 promoter. CAG-LSL-human Gq-coupled M3 muscarinic receptors (hM3Dq)-DREADD mice were crossed with CX3C chemokine receptor 1 (CX3CR1)-Cre mice, and immunohistochemistry images revealed that hM3Dq was selectively expressed on Iba1^+^ microglia, but not on astrocytes and neurons. Intrathecal (i.t.) administration of clozapine-N-oxide (CNO) elicited mechanical allodynia exclusively in male mice. Furthermore, the reactive microglia-dominant molecules that contributed to pain hypersensitivity in CX3CR1-hM3Dq were upregulated in mice of both sexes. The degree of upregulation was greater in male than in female mice. Depletion of spinal microglia using pexidartinib (PLX3397), a colony stimulating factor-1 receptor inhibitor, alleviated the male CX3CR1-hM3Dq mice from pain hypersensitivity and compromised the expression of inflammatory molecules. Thus, the chemogenetic activation of spinal microglia resulted in pain hypersensitivity in male mice, suggesting the sex-dependent molecular aspects of spinal microglia in the regulation of pain.

## 1. Introduction

Neuropathic pain (NP) is a debilitating condition caused by lesions or diseases in the peripheral or central nervous system (PNS or CNS) that results in unbearable pain for a long time [1]. Allodynia, hyperalgesia, and spontaneous pain are prominent symptoms in patients with NP, and conventional therapies such as opioids have a limited effect on NP [1,2]. NP affects approximately 7–10% of the general population and causes a huge socioeconomic burden [3,4]. To date, several animal models have been established to investigate the mechanisms of NP induction [5]. However, these remain unclear due to their complicated etiology. Therefore, there is a strong need to develop more specific and effective therapies that target the molecular aspects of NP.

Accumulating evidence suggests that glial cells are important in the pathogenesis of NP. Notably, microglia in the spinal dorsal horn (DH) play a critical role in the induction and maintenance of NP [6,7]. Under physiological conditions, resting microglia continuously survey the CNS microenvironment to protect neuronal functions [8,9]. However, events that threaten CNS homeostasis, such as nerve injury, trauma, or infection with pathogens, cause microglial proliferation and activation associated with morphological changes [7]. Reactive microglia often produce various pro-inflammatory cytokines and chemokines, including tumor necrosis factor α (TNF-α), interleukin 1β (IL-1β), C-C motif chemokine ligand 3 (CCL3), and CCL4, which are directly involved in the induction and maintenance of NP [10,11,12]. Traditionally, the majority of pain studies have been carried out in male animals, but recent findings suggest that there are sex differences in the neuron-glia communications that regulate pain. Sorge et al. demonstrated that spinal microglia are required for mechanical pain hypersensitivity following peripheral nerve injury in male mice, but not in female mice. This suggested sex-dependent roles of microglia in NP induction [13].

Designer receptors exclusively activated by designer drugs (DREADDs) are widely used for the selective manipulation of neuronal and cellular activity through typical G-protein-coupled receptor (GPCR) signaling pathways [14,15]. The activation of the human Gq-coupled M3 muscarinic receptors (hM3Dq) and Gi-coupled M4 muscarinic receptors (hM4Di) by clozapine-N-oxide (CNO) can induce Gq and Gi signaling, respectively [16]. DREADDs have been used to dissect several neurological events, such as pain, itching, and psychological behaviors, because they allow for the remote and non-invasive modulation of GPCR signaling pathways in a variety of neurons and glial cells in vivo. Recently, Grace et al. demonstrated that chemogenetic inhibition of spinal microglia by hM4Di attenuates mechanical allodynia following peripheral nerve injury, while chemogenetic activation of spinal microglia by hM3Dq induces mechanical allodynia in naïve rats using viral gene transfer [17]. Moreover, we also found that chemogenetic regulation of CX3C chemokine receptor 1 (CX3CR1)-expressing microglia using hM4Di exerts sex-dependent anti-allodynic effects in nerve injury—or paclitaxel-induced NP [18]. Nevertheless, current knowledge is not sufficient to fully understand the comprehensive roles of microglia leading to NP, and further investigations focusing on sex differences in microglial function would reveal the underlying mechanisms of NP.

In the present study, we aimed to determine whether there are sex differences in the behavioral and functional outcomes of chemogenetic activation of spinal microglia using Gq-DREADD driven by the microglia-specific Cx3cr1 promoter (CX3CR1-hM3Dq) in mice.

## 2. Materials and Methods

### 2.1. Mice

All animal experiments were approved by the Animal Research Committee of Wakayama Medical University and were carried out in accordance with the in-house guidelines for care and use of laboratory animals of Wakayama Medical University and the Ethical Guidelines of the International Association for the Study of Pain. CAG-LSL-Gq-DREADD mice [B6N;129-Tg(CAG-CHRM3*,-mCitrine)1Ute/J; stock #026220] and CX3CR1-Cre transgenic (Tg) mice [Tg(Cx3cr1-cre)MW126Gsat/Mmucd; stock #036395) were purchased from the Jackson Laboratory and Mutant Mouse Resource and Research Centers (MMRRC), respectively. CAG-LSL-hM3Dq-DREADD mice were maintained as heterozygous genotypes. For the Cre-dependent expression of the Gq-DREADD system in CX3CR1-expressing (CX3CR1^+^) cells, CAG-LSL-hM3Dq-DREADD mice were crossed with CX3CR1-Cre mice. Subsequently, 6−12-week-old mice heterozygous for hM3Dq and CX3CR1-Cre were used for the experiments. All mice were housed with a maximum of six in plastic cages at controlled temperature (23–24 °C) and humidity (60–70%), and the environment was maintained on a 12 h dark/light cycle, with free access to standard food and water.

### 2.2. Drug Administration

Clozapine N-oxide (CNO; Enzo Life Sciences, Farmingdale, NY, USA) was dissolved in sterile water and diluted as needed. CNO was administered intrathecally (i.t.) at a volume of 5 μL to isoflurane-anesthetized mice, as previously described [19]. Under isoflurane anesthesia, the mice were secured by a firm grip on the pelvic girdle, and drugs were injected by lumbar puncture between the L5 and L6 vertebrae using a 30-gauge needle fitted to a Hamilton microsyringe. CNO was also administered intraperitoneally (i.p.) at a volume of 0.1 mL/10 g body weight to awake mice.

### 2.3. Microglia Depletion

To deplete microglia in vivo, pexidartinib (PLX3397; MedChemExpress, NJ, USA), a colony-stimulating factor 1 receptor (CSF-1R) inhibitor, was formulated into the AIN-76A rodent diet (Research Diets, NJ, USA) at 290 mg/kg chow and administered to mice for 10 days. The dose of PLX3397 and its route of administration were determined according to previously published studies [20,21]. The AIN-76A rodent diet was used as control.

### 2.4. Immunohistochemistry

The mice were deeply anesthetized with pentobarbital (100 mg/kg, i.p.) and transcardially perfused with ice-cold phosphate-buffered saline (PBS), followed by a 4% (*w/v*) paraformaldehyde/phosphate buffer solution. Then, the lumbar spinal cord (L4–5) was dissected and after post-fixation in 4% paraformaldehyde/ phosphate buffer solution, it was placed overnight in a 30% (*w/v*) sucrose/PBS solution at 4 °C for cryoprotection. The tissue was then embedded in a freezing optimal cutting temperature compound (Sakura, Tokyo, Japan). Subsequently, the specimens were longitudinally cut into 30 μm thick sections using a cryostat (Leica Microsystems, Wetzlar, Germany). The sections were treated with PBS containing 0.1% Triton X-100 (PBST) for 1 h and then blocked with Blocking One Histo (Nacalai Tesque, Inc., Kyoto, Japan) at 15–25 °C for 5–10 min. They were then incubated with primary antibodies against hemagglutinin (HA) epitope-tag (mouse monoclonal, 1:250; BioLegend, San Diego, CA, USA), ionized calcium-binding adaptor protein-1 (Iba1; rabbit polyclonal, 1:500; Wako, Japan), glial fibrillary acidic protein (GFAP; rabbit polyclonal, 1:500; Proteintech, Rosemont, IL, USA), and neuronal nuclei (NeuN; rabbit monoclonal, 1:500; Millipore, Billerica, MA, USA) at 4 °C overnight. Subsequently, the sections were rinsed in PBST and incubated with fluorescence-conjugated secondary antibodies (1:200; Abcam, Cambridge, UK) at 15–25 °C for 1 h. They were then rinsed in PBS and incubated with Hoechst 33342 (1:1000; Invitrogen, Carlsbad, CA, USA) for 10 min at room temperature in the dark. Finally, the sections were washed with PBS (10 min), mounted on glass slides, and covered with a cover slip using PermaFluor (Thermo Fisher Scientific) as the mounting medium. Fluorescence images were obtained using a confocal laser scanning microscope (Carl Zeiss, Oberkochen, Germany). The number of hypertrophied Iba1-positive cells were measured in an indicated square of 200 × 200 μm^2^ area using ImageJ software.

### 2.5. Von Frey Test

To evaluate mechanical allodynia, the 50% paw withdrawal threshold was determined using the von Frey test, in accordance with a previously described method [18,22]. Briefly, the mice were individually placed on a metal mesh (5 × 5 mm) grid floor and covered with an acrylic box. Before the test, the mice were habituated to the experimental environment for at least 3−4 h. On the test day, after adaptation for 2–3 h, calibrated von Frey filaments (North Coast Medical, Inc., Gilroy, CA, USA) were applied to the middle of the plantar surface of the hind paw through the bottom of the mesh floor. The filament set used in this study consisted of nine calibrated von Frey filaments: 0.02, 0.04, 0.07, 0.16, 0.4, 0.6, 1.0, 1.4, and 2.0 g. In the paradigm of the up–down method, the test always started with the application of 0.4 g filaments. Quick withdrawal, shaking, biting, or licking of the stimulated paw were regarded as positive paw withdrawal responses. In the absence of a paw withdrawal response to the selected force, the next stronger stimulus was applied. The next weaker stimulus was chosen in the presence of paw withdrawal. In accordance with Chaplan’s procedure, upon crossing the response threshold for the first time (the two responses straddling the threshold), four additional stimuli were applied. Based on the responses to the von Frey filaments series, the 50% paw withdrawal threshold was calculated according to the method described by Dixon [23].

### 2.6. Hargreaves Test

To evaluate thermal hyperalgesia, the withdrawal threshold was determined using the Hargreaves test as previously described (PMID: 25630024). The mice were placed on a glass sheet and covered with a clear acrylic box. After adaptation for 2–3 h, a radiant heat source (IITC 390 Plantar Test Analgesia Meter, Neuroscience) was positioned under the glass sheet and applied to the plantar surface of the both hind paws. Withdrawal latencies were evaluated on the basis of the mean latency of three stimulations. A cutoff latency of 15 s was set to avoid tissue damage.

### 2.7. Reverse Transcription-Quantitative Polymerase Chain Reaction (RT-qPCR)

Mice were euthanized by decapitation after 24 h of the drug administration. The fresh lumbar spinal cord samples were collected in RNAlater solution (Thermo Fisher Scientific, Waltham, MA, USA). The TRIzol Plus RNA purification kit (Thermo Fisher Scientific) was used to isolate total RNA from tissues following the manufacturer’s instructions. The spinal cord was placed in a 1.5 mL RNase-free tube and homogenized with TRIzol reagent. Chloroform was added to each sample and mixed gently, which was then centrifuged at 4 °C for 15 min. The aqueous phase containing RNA was transferred to a fresh tube, and RNA was isolated using a purification column. Total RNA extract (1 µg) was used to synthesize cDNA by reverse transcription (RT) as follows. Total RNA was incubated with random primers (Promega, Madison, WI, USA) at 70 °C for 5 min and then cooled on ice. Samples were converted to cDNA by incubation with M-MLV Reverse Transcriptase (Promega) and dNTPMix (Promega) at 37 °C for 60 min. Quantitative polymerase chain reaction (qPCR) was performed using an ECO real-time PCR system (AS One, Osaka, Japan) with template cDNA (10 ng) primers for each gene (Thermo Fisher Scientific) and SYBR Premix Ex Taq II (Takara Bio Inc., Kusatsu, Japan). The primer sequences are listed in Table 1. Reactions were performed under the following conditions: 10 min at 95 °C, followed by 50 cycles of step two that comprised 10 s at 95 °C, and 30 s at 60 °C. Fluorescence intensities were recorded, and the data were normalized to glyceraldehyde-3-phosphate dehydrogenase (GAPDH).

### 2.8. Statistical Analysis

Data are presented as the mean ± standard error of the mean (SEM). Statistical analyses were performed using Student’s t-test and one-way analysis of variance (ANOVA) followed by Tukey’s multiple comparison test, or by a two-way ANOVA followed by Bonferroni’s multiple comparison test, as appropriate. Statistical significance was set at *p* < 0.05.

## 3. Results

**Figure 1:**
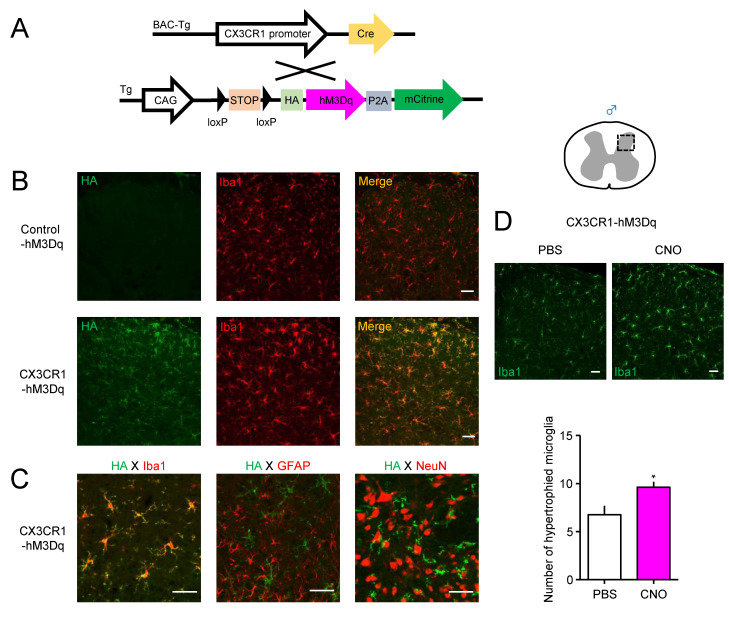
Activation of spinal microglia by clozapine-N-oxide (CNO) in male CX3C chemokine receptor 1, human Gq-coupled M3 muscarinic receptors (CX3CR1-hM3Dq) mice. (**A**) Scheme of Cre-dependent expression of hM3Dq in CX3CR1-expressing (CX3CR1^+^) cells by crossing CX3CR1-Cre (Tg) mice with CAG-LSL-Gq-designer receptors exclusively activated by designer drugs (Gq-DREADD) mice (Control-hM3Dq). (B–D) CNO (2 nmol) was intrathecally (i.t.) administered to male naïve Control-hM3Dq and CX3CR1-hM3Dq mice, and immunohistochemistry was performed one day after administration. (**B**) The Cre-dependent expression of hemagglutinin (HA)-tagged hM3Dq on the spinal dorsal horn (DH) in Control-hM3Dq and CX3CR1-hM3Dq mice. (**C**) Localization of HA-hM3Dq in Iba1^+^ microglia, but not GFAP^+^ astrocytes or NeuN^+^ neurons. (**D**) Representative micrographs and the number of Iba1^+^ microglia within the square of 200 × 200 μm^2^ in the spinal DH in male CX3CR1-hM3Dq mice administered phosphate-buffered saline (PBS) or CNO. Data are presented as mean ± standard error of the mean (SEM). *n* = 4–8. *, *p* < 0.05. The square in the spinal cord shows the region of micrographs. Scale bars = 40 μm.

For the Cre-dependent expression of hM3Dq in CX3CR1^+^ cells, CAG-LSL-hM3Dq-DREADD (Control-hM3Dq) mice were crossed with CX3C chemokine receptor 1 (CX3CR1)-Cre mice (Figure 1A). Following Cre-mediated removal of an upstream floxed-STOP cassette, the expression of HA-tagged hM3Dq was observed using an antibody against HA by immunohistochemistry. A day after i.t. administration of CNO (2 nmol), HA-hM3Dq was highly expressed in the spinal DH of CX3CR1-Cre/CAG-LSL-hM3Dq-DREADD (CX3CR1-hM3Dq) mice, but not in Control-hM3Dq mice (Figure 1B). Moreover, in male CX3CR1-hM3Dq mice, HA-hM3Dq completely overlapped with the microglial marker Iba1, whereas it did not colocalize in GFAP^+^ astrocytes or NeuN^+^ neurons, indicating that the expression of hM3Dq was restricted to spinal microglia within the spinal DH (Figure 1C). CNO administration upregulated Iba1 expression, and the number of Iba1^+^ microglia were increased in the spinal DH of male CX3CR1-hM3Dq mice compared to PBS administration (Figure 1D). Similarly, HA-hM3Dq was also expressed in Iba1^+^ microglia in the spinal DH of female CX3CR1-hM3Dq mice, but not in female Control-hM3Dq mice (Figure S1).

**Figure 2:**
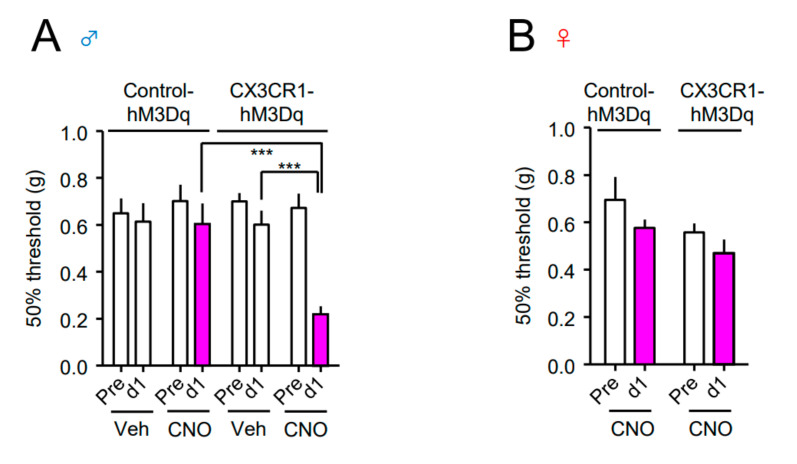
Induction of mechanical allodynia by CNO in male CX3CR1-hM3Dq mice. (**A**,**B**) CNO (2 nmol) or vehicle (Veh) was intrathecally (i.t.) administered to naïve Control-hM3Dq and CX3CR1-hM3Dq mice of both sexes (**A**; male, **B**; female). The 50% paw withdrawal threshold was assessed by the up–down method using the von Frey test before (pre) and one day after administration. Data are presented as mean ± SEM. (A) *n* = 5−9. (B) *n* = 6−8. ***, *p* < 0.001.

**Figure 3:**
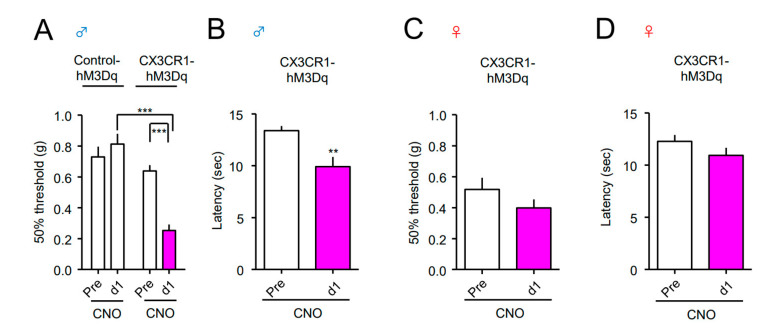
Induction of mechanical allodynia and thermal hyperalgesia by systemic CNO in male CX3CR1-hM3Dq mice. CNO (1 mg/kg) was intraperitoneally (i.p.) administered to naïve Control-hM3Dq and CX3CR1-hM3Dq mice of both sexes (**A**,**B**; male, **C**,**D**; female). The 50% paw withdrawal threshold (**A**,**C**) or withdrawal latency (**B**,**D**) was assessed by the von Frey test or the Hargreaves test, respectively, before and one day after administration. Data are presented as mean ± SEM. (**A**) *n* =10−14. (**B**) *n* = 12. (**C**) *n* = 6. (**D**) *n* = 11. ***, *p* < 0.001; **, *p* < 0.01.

Next, we investigated the effect of Gq-DREADD in the spinal CX3CR1^+^ microglia on pain sensitivity. CNO (2 nmol) or vehicle (Veh) was i.t. administered to both the Control-hM3Dq and CX3CR1-hM3Dq mice, and the von Frey test was performed before (pre) and one day after administration. A single administration of CNO significantly reduced the mechanical pain threshold, indicating mechanical allodynia in male CX3CR1-hM3Dq mice, but not in male Control-hM3Dq mice (Figure 2A). In contrast, CNO administration did not elicit mechanical allodynia in female CX3CR1-hM3Dq mice (Figure 2B). Consistently, a single i.p. administration of CNO (1 mg/kg) elicited mechanical allodynia and thermal hyperalgesia in male CX3CR1-hM3Dq mice, but not in male Control-hM3Dq mice and female CX3CR1-hM3Dq mice (Figure 3A-D). 

**Figure 4:**
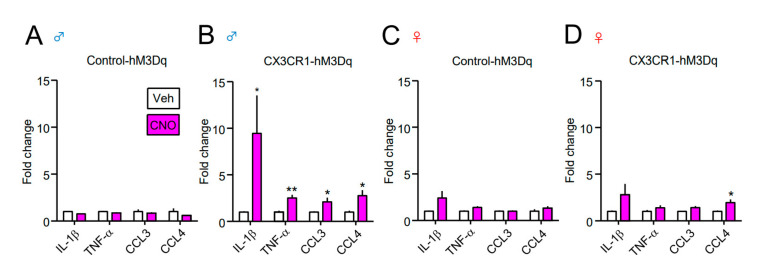
Upregulation of inflammatory mediators in the spinal cord of male CX3CR1-hM3Dq mice. CNO (2 nmol) or Veh was i.t. administered to naïve Control-hM3Dq and CX3CR1-hM3Dq mice of both sexes (**A**,**B**; male, **C**,**D**; female), and the spinal cord was collected one day after administration. The mRNA expression of inflammatory mediator (interleukin 1β (IL-1β), tumor necrosis factor α (TNF-α), C-C motif chemokine ligand 3 (CCL3), and CCL4) was analyzed by RT-qPCR. Data are presented as mean ± SEM. *n* = 6−9. **, *p* < 0.01. *, *p* < 0.05. vs. Veh.

**Figure 5:**
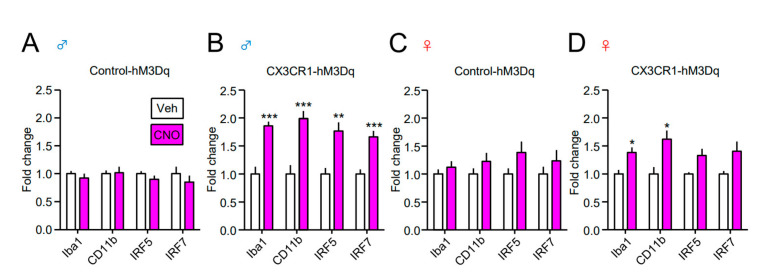
Upregulation of microglial markers in the spinal cord of male CX3CR1-hM3Dq mice. CNO (2 nmol) or Veh was i.t. administered to naïve Control-hM3Dq and CX3CR1-hM3Dq mice of both sexes (**A**,**B**; male, **C**,**D**; female), and the spinal cord was collected one day after administration. The mRNA expression of microglial markers (Iba1, CD11b, interferon regulatory factor-5 (IRF5) and IRF7) was analyzed by RT-qPCR. Data are presented as mean ± SEM. *n* = 6−9. ***, *p* < 0.001; **, *p* < 0.01; *, *p* < 0.05. vs. Veh.

To determine whether Gq-DREADD selectively modulates the microglial function, we assessed the mRNA expression levels of reactive microglia-dominant molecules. CNO (2 nmol) or Veh was i.t. administered to both the Control-hM3Dq and CX3CR1-hM3Dq mice, and RT-qPCR was performed one day after administration (Figure 4 and Figure 5). A single i.t. administration of CNO upregulated the expression of inflammatory mediators (IL-1β, TNF-α, CCL3, and CCL4) in the spinal cord of male CX3CR1-hM3Dq mice compared to male Control-hM3Dq mice (Figure 4A,B). On the other hand, CNO slightly increased the expression of these inflammatory mediators in female CX3CR1-hM3Dq mice, but this was clearly lower than that in male CX3CR1-hM3Dq mice (Figure 4D).

In addition, CNO administration markedly upregulated the expression of microglial markers (Iba1 and CD11b) and reactive microglial factors (interferon regulatory factor-5 (IRF5) and IRF7) in male CX3CR1-hM3Dq mice compared to the control-hM3Dq male mice (Figure 5A,B). Notably, CNO-induced upregulation of such microglial factors was also observed in female CX3CR1-hM3Dq mice. Nevertheless, the degree of such upregulation in female CX3CR1-hM3Dq mice was lower than that in male CX3CR1-hM3Dq mice. Consistent with i.t. administration, the i.p. administration of CNO also upregulated the expression of inflammatory mediators (IL-1β, CCL3, and CCL4) and microglial factors (Iba1 and IRF5) in the spinal cord of male CX3CR1-hM3Dq mice (Figure S2).

**Figure 6:**
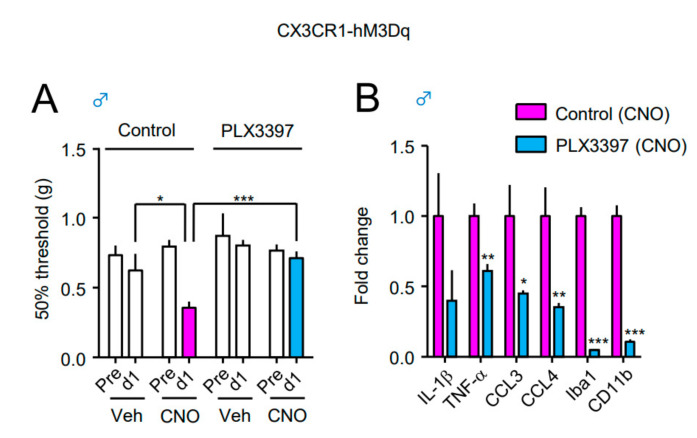
Contribution of microglia to CNO-induced mechanical allodynia and upregulation of inflammatory molecules in male CX3CR1-hM3Dq mice. Mice were fed with a control diet or pexidartinib (PLX3397), a colony-stimulating factor 1 (CSF-1) receptor inhibitor, diet for 10 days, and CNO (2 nmol) or Veh was i.t. administered on the ninth day. (**A**) The 50% paw withdrawal threshold was assessed by the up–down method using the von Frey test before (day 9; pre) and one day after administration (day 10; d1). (**B**) The mRNA expression of inflammatory mediators (IL-1β, TNF-α, CCL3, and CCL4) and microglial markers (Iba1, CD11b) in the spinal cord was analyzed by RT-qPCR one day after CNO administration. Data are presented as mean ± SEM. (**A**) *n* = 5−10. (**B**) *n* = 9−10. ***, *p* < 0.001. **, *p* < 0.01; *, *p* < 0.05. vs. (**B**) control diet.

Finally, to further demonstrate the direct contribution of microglia to the CNO-induced upregulation of inflammatory molecules and mechanical allodynia in male CX3CR1-hM3Dq mice, PLX3397, a CSF-1R inhibitor that depletes CNS microglia, was orally administered to mice for 10 days. CNO (2 nmol) or Veh was i.t. administered on the ninth day, and the von Frey test was carried out before (pre) and one day after administration. Subsequently, mRNA expression of inflammatory mediators (IL-1β, TNF-α, CCL3, and CCL4) and microglial markers (Iba1 and CD11b) in the spinal cord was evaluated. CNO-induced mechanical allodynia in male CX3CR1-hM3Dq mice that were fed a control diet was completely prevented by PLX3397 treatment (Figure 6A). Furthermore, CNO-induced upregulation of microglia-dominant molecules in male CX3CR1-hM3Dq mice was also significantly decreased upon PLX3397 treatment (Figure 6B), suggesting that these factors are derived from reactive microglia in the spinal cord.

## 4. Discussion

Emerging evidence suggests that microglial activation is a fundamental component for the pathogenesis of CNS diseases that are associated with neuroinflammation [24,25,26]. With regards to NP, microglial activation persists for several weeks following peripheral nerve injury, and reactive microglia often produce several inflammatory molecules that result in long-lasting neuroinflammation [7]. Among various soluble factors upregulated after nerve injury, CSF-1 is a key regulator of the proliferation, differentiation, and survival of spinal microglia, as CSF-1R is only expressed in the microglia within the spinal DH [27,28]. A recent study demonstrated that CSF-1 is produced from the injured primary sensory neurons and then acts on spinal microglia, contributing to the development of NP [20,27]. After nerve injury, pro-inflammatory cytokines (e.g., IL-1β and TNF-α) and chemokines (e.g., CCL3 and CCL4) are produced by reactive microglia, which modulate pain sensitivity through their cognate receptors expressed in pain-processing neurons and glial cells within the spinal DH [10,29,30,31]. Indeed, i.t. administration of these cytokines and chemokines elicited pain hypersensitivity, while i.t. administration of their inhibitors prevented NP following nerve injury in rodents [32,33,34,35]. Thus, it has been generally considered that microglial activation plays a pivotal role in the pathogenesis of NP.

Despite numerous studies in the field of pain, careful attention is required for the interpretation of microglial functions due to the technical difficulty of specifically targeting microglia without affecting other cells. In fact, minocycline, a commonly used microglial inhibitor, relieves NP, but is also known to act on both microglia and neurons [36,37,38]. Although several studies have reported on the relieving effects of microglial inhibitors (i.e., minocycline and propentofylline) [6,39,40], there has been a lack of direct evidence that indicated an implicit role of microglia in NP induction by selectively manipulating spinal microglia. To investigate whether selective activation of spinal microglia affects neuroinflammation associated with pain hypersensitivity, we used CX3CR1-hM3Dq mice that express hM3Dq in a Cre-dependent manner under the microglia-specific promoter CX3CR1 in the CNS. Recently, it was revealed that the activation of spinal microglia by CD68 promoter-driven Gq-DREADD induced mechanical allodynia in rats [17]. In addition, the induction of Gq-DREADD in murine microglial cells upregulated inflammatory cytokines such as IL-1β and TNF-α, with elevated intracellular calcium levels [41]. Consistent with these findings, we found that the activation of spinal microglia by Gq-DREADD caused mechanical allodynia and the upregulation of inflammatory mediators (IL-1β, TNF-α, CCL3, and CCL4), microglial markers (Iba1 and CD11b), and inflammatory microglial factors (IRF5 and IRF7) within the spinal cord. These lines of evidence indicate that transient activation of microglia by Gq-DREADD after a single dose of CNO increases cytokine expression in the CNS, leading to mechanical allodynia.

The IRF family of transcription factors has received much attention for the regulation of innate immune cell development and responses. IRF5 is widely expressed in several hematopoietic cells, including monocytes/macrophages and dendritic cells in the periphery [42,43]. Besides, IRF5 induces the expression of inflammatory cytokines, such as TNF-α, whereas IRF5 deficiency results in lower levels of inflammatory molecules [44]. Masuda et al. demonstrated that IRF5 is a key regulator that induces reactive microglia underlying NP after peripheral nerve injury [45]. Moreover, IRF5 largely contributes to the overexpression of pain-related inflammatory cytokines and chemokines under pathological conditions in the CNS [45,46]. It has also been reported that both IRF5 and IRF7 play pivotal roles in the pathogenesis of CNS diseases with microglial activation [47]. Given that IRFs are crucial for the induction of the inflammatory state in microglia, hM3Dq signaling might induce reactive microglia to be closely related to pain hypersensitivity. Notably, mechanical allodynia and the upregulation of inflammatory molecules by the activation of Gq-DREADD were prevented by the depletion of microglia using the CSF-1R inhibitor, PLX3397. These results strongly suggest that the Gq-DREADD system was clearly restricted in the spinal microglia, but not in other pain-modulating cells.

It is well known that microglia and astrocytes play important roles in the induction, development, and maintenance of pain [7,48,49]. However, the majority of such evidence was obtained from studies using male mice, but not female mice [50]. To date, the effects of glial cell activation on pain hypersensitivity in females are not fully understood. Importantly, Sorge et al. found that i.t. injection of minocycline suppressed NP in male mice, but not in female mice. The i.t. injection of the microglia-targeting toxin also exerted significant inhibitory effects on mechanical allodynia following nerve injury in male mice, but not in female mice. Moreover, inhibitors of key signaling molecules in the spinal cord microglia-to-neuron pain pathway also blocked mechanical allodynia following nerve injury in males, but not in female mice [13]. These results suggest that reactive microglia elicit pain hypersensitivity in male mice, but not female mice. In agreement with this, Chen et al. found that i.t. injection of the microglia activator caspase 6 protein elicited mechanical hypersensitivity only in naïve male mice, but not in females [51,52]. Our results also clearly showed that spinal microglial activation via hM3Dq elicited mechanical allodynia in a sex-dependent manner. Nevertheless, it should be emphasized that the induction of Gq-DREADD did not significantly activate spinal microglia in female CX3CR1-hM3Dq mice, as was evident from the RT-qPCR analysis. Thus, there might be big differences in character and sensitivity to several stimuli in microglia between males and females.

Growing evidence suggests that CNS microglia show sex-specific features defined by transcriptomic and functional analyses [53]. Expression patterns of inflammatory molecules in brain microglia are significantly sex-dependent; male microglia are neuroinflammatory, while female microglia are neuroprotective [54]. Given that estrogens suppress neuroinflammatory responses [54], it might be a key determinant for the neuroprotective feature of female microglia. Notably, transplantation of brain microglia isolated from female mice clearly attenuated damages after ischemic stroke in male mice [54], indicating that exposure to sex hormones may largely affect sex-specific features of CNS microglia. Unlike males, female have an ovarian cycle that determines estrogen levels in the body, and several reports indicate that estrogens modulate the pain threshold in humans and rodents [55,56]. Although pain hypersensitivity after nerve injury is similarly observed between both sexes [57], it is pivotal to investigate whether fluctuations of estrogen levels regulate intractable pain associated with microglial activation. Recently, Yu et al. demonstrated that CSF-1 derived from primary sensory neurons after nerve injury not only activated spinal microglia, but also increased in peripheral macrophages in the dorsal root ganglia (DRG) in male mice [58]. Interestingly, fewer contributions of CSF-1 to the expansion of DRG macrophages were observed in female mice, despite the fact that the selective depletion of DRG macrophages relieved the nerve injury-induced mechanical hypersensitivity caused by DRG macrophages in both male and female mice [58]. If pathophysiological roles of peripheral macrophages in NP are sex hormone-independent, unlike microglia, the exploration of regulating mechanisms for peripheral macrophages is not only scientifically interesting, but also clinically important.

To assess the selective roles of reactive microglia, distinct groups of researchers have demonstrated that Gi-DREADD can also be used for the inhibition of spinal microglia under NP. Our previous findings clearly indicate that the induction of Gi-DREADD in CX3CR1^+^ microglia significantly attenuates mechanical allodynia caused by peripheral nerve injury or paclitaxel administration [18]. Similarly, Grace et al. demonstrated that the induction of Gi-DREADD in spinal microglia temporarily relieved mechanical allodynia after nerve injury in vivo, and attenuated inflammatory responses by lipopolysaccharide treatment in vitro [17]. Recently, Yi et al. also showed that the induction of Gi-DREADD in microglia suppressed microglial activation and pain hypersensitivity after nerve injury [59]. These lines of evidence share a common view that Gi-DREADD in reactive microglia might attenuate pain hypersensitivity caused by nerve injury. Similar to typical inhibitors for spinal microglia, the relieving effects of Gi-DREADD in microglia on NP were observed in only male mice, but not in female mice, as reported previously. It is worth noting that Gq- and Gi-DREADD can bidirectionally modulate microglial activity in vivo, and are useful to explore the pathophysiological roles of spinal microglia. However, in contrast to our results, Yi et al. demonstrated that the activation of Gi-DREADD in spinal microglia attenuated NP in both male and female mice [59]. Since there is some discrepancy among the research communities, further evidence is required to fully understand the sex-dependent roles of spinal microglia in pain regulation.

Collectively, we clarified that the induction of Gq-DREADD in spinal CX3CR1^+^ microglia elicited mechanical allodynia in male mice, but not in female mice. The hM3Dq signaling following the CNO administration enhanced the expression of the reactive microglia-dominant molecules in the spinal cord, leading to pain hypersensitivity in male CX3CR1-hM3Dq mice. Notably, we also found that the effects of Gq-DREADD on spinal microglia in male mice were significantly greater than those in female mice, which is consistent with the behavioral outcomes. To the best of our knowledge, this is an important study to demonstrate the sex differences in microglial regulation of pain hypersensitivity based on the chemogenetic activation of CX3CR1^+^ microglia in an in vivo model. Our studies using the DREADD systems support the notion that microglia play a crucial role in pain regulation in male mice, but there is a strong need to reveal the sex-dependent regulation of microglia to generate novel therapeutic approaches for NP in the future.

## Figures and Tables

**Table 1:** Primer sequences for reverse transcription-quantitative polymerase chain reaction (RT-qPCR).

Gene	Forward (5′ to 3′)	Reverse (5′ to 3′)
GAPDH	GGGTGTGAACCACGAGAAAT	ACTGTGGTCATGAGCCCTTC
IL-1β	AAAGCTCTCCACCTCAATGG	AGGCCACAGGTATTTTGTCG
TNF-α	CCCCAAAGGGATGAGAAGTT	TGGGCTACAGGCTTGTCACT
CCL3	CTGCCCTTGCTGTTCTTCTC	GTGGAATCTTCCGGCTGTAG
CCL4	ATGAACTCTGCGTGTCTGC	GCCGGGAGGTGTAAGAGAAA
Iba1	ATGAGCCAAAGCAGGGATTT	TTGGGATCATCGAGGAATTG
CD11b	GTTTCTACTGTCCCCCAGCA	GTTGGAGCCGAACAAATAGC
IRF5	ACACTGAAGGGGTGATGAG	CGAGGGCCATCATAGAACAG
IRF7	GTGTGTCCCCAGGATCATTT	CTGCAGAACCTGAAGCAAGA

## Data Availability

The data presented in this study are available on request from the corresponding author.

## Supplementary Materials

The following are available online at https://www.mdpi.com/article/10.3390/cells10040874/s1, Figure S1: Expression of hM3Dq in spinal microglia of female CX3CR1-hM3Dq mice.
