# Supplementary material for: Chemogenetic Activation of CX3CR1-Expressing Spinal Microglia Using Gq-DREADD Elicits Mechanical Allodynia in Male Mice
Source: Cells. 2021 Apr 12;10(4):874. doi: 10.3390/cells10040874 (PMC8069983; doi:10.3390/cells10040874)

Supplementary Materials:

Fig. S1 Expression of hM3Dq in spinal microglia of female CX3CR1-hM3Dq mice.

The Cre-dependent expression and localization of HA-hM3Dq in Iba1⁺ microglia in the spinal DH of female Control-hM3Dq and CX3CR1-hM3Dq mice was visualized by immunohistochemistry. The square shows the region of the micrographs. Scale bars = 40 μm.

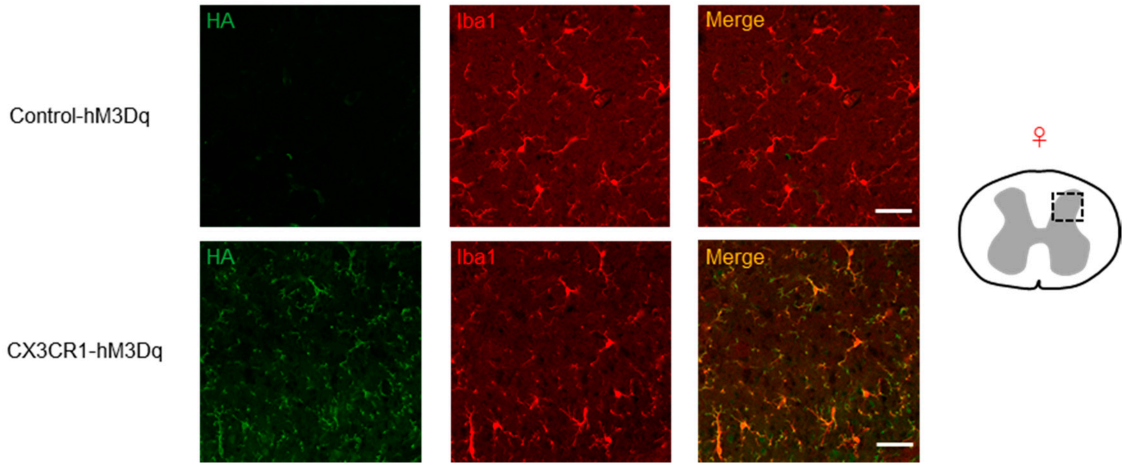

Fig. S2 Upregulation of inflammatory mediators and microglial markers in the spinal cord of male CX3CR1-hM3Dq mice. CNO (1 mg/kg) was i.p. administered to naïve male Control-hM3Dq and CX3CR1-hM3Dq mice. The mRNA expression of inflammatory mediators (IL-1β, CCL3 and CCL4) and microglial molecules (Iba1, IRF5 and IRF7) in the spinal cord one day after CNO administration was analyzed by RT-qPCR. Data are presented as mean ± S.E.M. n = 6-10. ***, P < 0.001; **, P < 0.01; *, P < 0.05. vs. Control-hM3Dq.

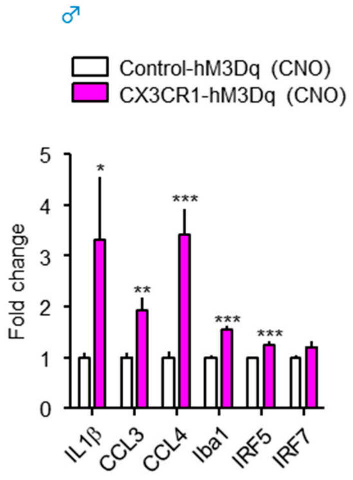

Supplement: Supplementary file 1 [file cells-10-00874-s001.pdf]
